# Therapeutic Immunomodulation of Tumor-Lymphatic Crosstalk via Intratumoral Immunotherapy

**DOI:** 10.1021/acs.molpharmaceut.4c00692

**Published:** 2024-10-31

**Authors:** Samuel N. Lucas, Susan N. Thomas

**Affiliations:** † Wallace H. Coulter Department of Biomedical Engineering, Georgia Institute of Technology and Emory University, Atlanta, Georgia 30332, United States of America; ‡ George W. Woodruff School of Mechanical Engineering, Georgia Institute of Technology, Atlanta, Georgia 30332, United States of America; § Parker H. Petit Institute for Bioengineering and Bioscience, Georgia Institute of Technology, Atlanta, Georgia 30332, United States of America; ∥ Winship Cancer Institute, Emory University, Atlanta, Georgia 30322, United States of America

**Keywords:** sentinel lymph node, lymphatics, immune crosstalk, cancer immunotherapy, drug delivery

## Abstract

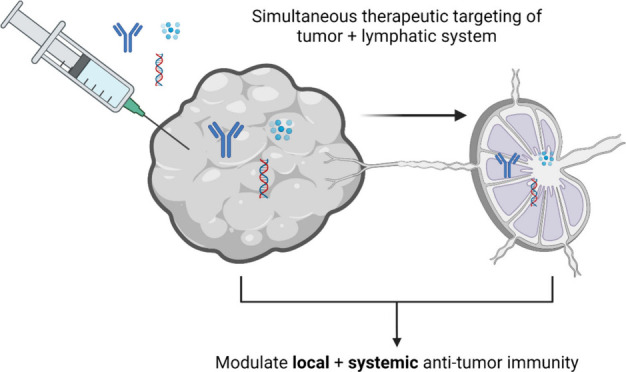

Intra- and peritumoral lymphatics and tumor-draining lymph nodes play major roles in mediating the adaptive immune response to cancer immunotherapy. Despite this, current paradigms of clinical cancer management seldom seek to therapeutically modulate tumor-lymphatic immune crosstalk. This review explores recent developments that set the stage for how this regulatory axis can be therapeutically manipulated, with a particular emphasis on tumor-localized immunomodulation. Building on this idea, the nature of tumor-lymphatic immune crosstalk and relevant immunotherapeutic targets and pathways are reviewed, with a focus on their translational potential. Engineered drug delivery systems that enhance intratumoral immunotherapy by improving drug delivery to both the tumor and lymph nodes are also highlighted.

## 1 Introduction

Sentinel lymph node (SLN) status has been a mainstay of clinical cancer diagnoses and prognoses for the better part of a century; only more recently have the numerous regulatory roles that the crosstalk between the tumor microenvironment and the lymphatic system play in disease progression come into focus.^1−3^ These roles include not only providing a pathway for spread of disease from regional to systemic but also developing an antitumor adaptive immune response, facilitated by peritumoral lymphatics, locally as well as in tumor-draining lymph nodes (TDLNs), that is necessary for tumor immune surveillance and elimination.^1,4,5^ Critically, these TDLNs are sites where systemically active immune responses can be modulated. Tumors can also coopt immune regulatory pathways to modulate immune activity in the TDLN and peritumoral lymphatics in order to evade immune surveillance and destruction.^6^ These immune regulatory effects are important factors limiting the effects of cancer immunotherapies. The transformative success of immunotherapies in the past decade thus stands to further benefit from expanding targets to include those implicated in tumor-lymphatic immune crosstalk.^7−9^

The clinical advances in cancer immunotherapy have been accompanied by an explosion of preclinical and clinical investigations into new immunotherapeutic modalities and targets. Yet most immunotherapies currently in clinical use continue to be administered systemically, despite the nature of the adaptive immune system allowing localized immune education that yields systemic immunity. Systemically administered therapies have relatively poor access to the tumor. Even purportedly tumor-targeting antibodies such as antibody-drug conjugates (ADCs) see less than 1% of the administered dose reach the tumor following intravenous injection.^10,11^ Access to peritumoral lymphatics, SLNs, and TDLNs from systemic administration is even poorer, making it easy to understand how therapeutic immunomodulation within the regional lymphatic bed of solid tumors has long been overlooked. A potential solution to improving the accumulation of therapeutic payload in both tumors and their lymphatic basins is suggested by a commonly used diagnostic technique: SLN mapping is routinely achieved by intratumoral or peritumoral injection of contrast agent. Treating solid tumors regionally can allow high levels of drug delivery to tumors and TDLNs as an alternative to systemic administration. In the context of immunotherapy, such approaches could modulate both local immunity (in the tumor) and systemic immunity (via the TDLNs), not only controlling the treated tumor but also generating systemic control of disease despite the treatment being regionally applied. Indeed, such a therapeutic strategy may be key to overcoming the historical failure of intratumoral therapy in adequately treating distant metastases.

Developments in diagnostic techniques and treatment protocols in recent years increasingly fortify the relevance of peritumoral lymphatics and the SLN as therapeutic targets. For example, lymphatic mapping, which uses a tracer to identify the LN(s) draining a tumor basin, is an ever more widely used diagnostic tool that could also plausibly enable deliberate therapeutic targeting of the SLN. Complete LN dissection (CLND), which is the preemptive surgical removal of all LNs locoregional to an identified tumor, has dramatically decreased in prevalence due to mounting evidence that routine CLND does not benefit most patients. For example, randomized phase 3 clinical trials in melanoma have indicated no difference in survival for patients with SLN metastasis who receive CLND compared to observation.^12,13^ Similarly, despite its potential diagnostic value, even routine SLN biopsy (SLNB) has not resulted in significantly improved disease-specific survival versus observation in melanoma or breast cancer.^14−16^ As such, debate continues over whether LN metastasis occurs in advance or concurrently with systemic metastasis. Thus, now and looking to the future, more LNs are being left in patients post diagnosis, offering unique, heretofore untapped opportunities for immunomodulation.

**Figure 1:**
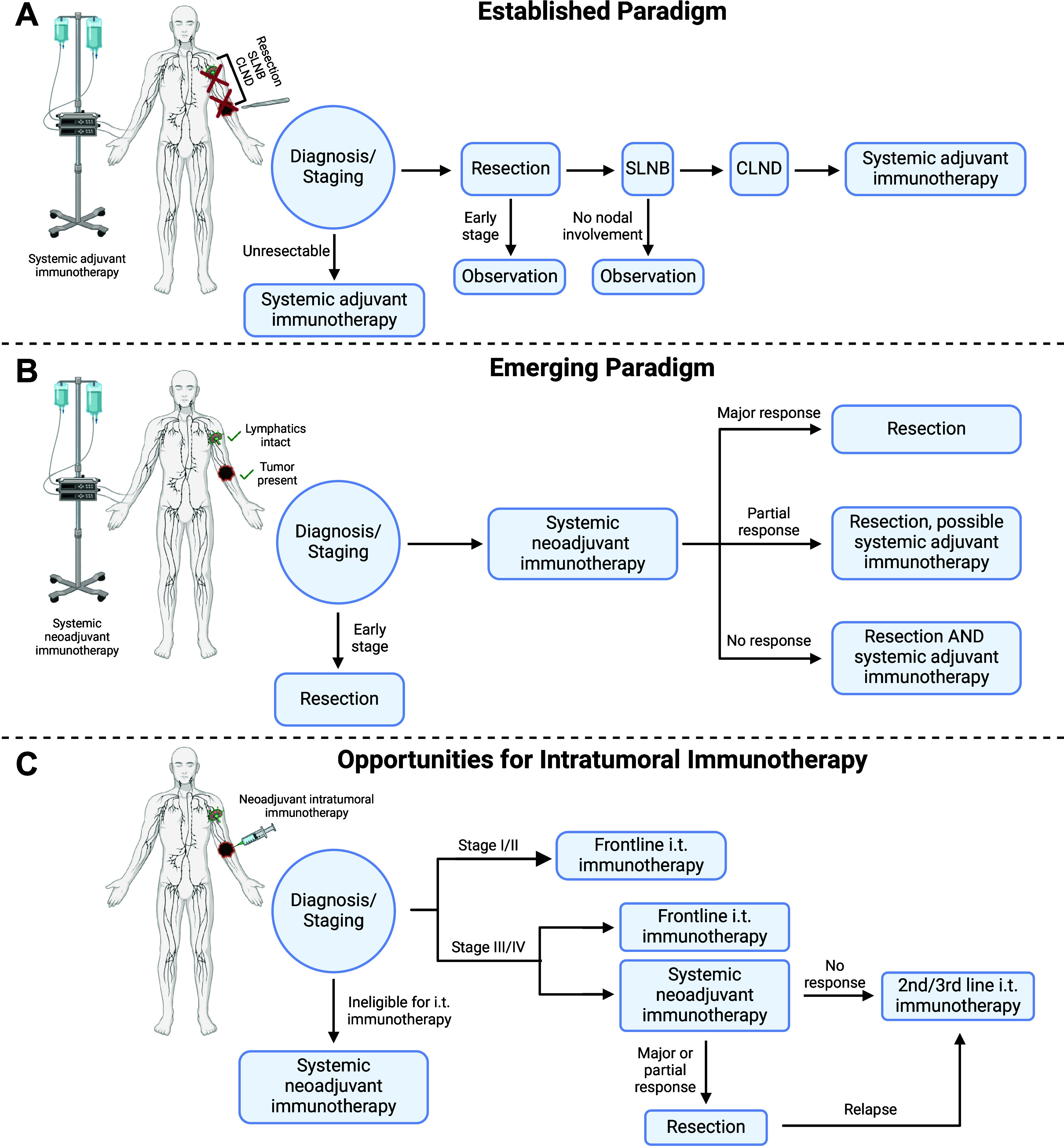
Evolving clinical paradigms increasingly leave tumor and lymphatics intact at the time of first immunotherapeutic treatment, creating opportunities for intratumoral immunotherapy. The following examples are based primarily around changes in melanoma treatment paradigms but are emblematic of changes more broadly in cancer treatment as well. Representative examples of A) the established paradigm for surgery and immunotherapy sequencing including CLND/SLNB; B) the emerging paradigm for surgery and immunotherapy sequencing with adjuvant options dependent on neoadjuvant response; C) opportunities for intratumoral immunotherapy to interface with the emerging immunotherapeutic paradigm by modulating tumor-lymphatic crosstalk.

In parallel to changes in clinical practices around LN management/removal, treatment paradigms have changed (Figure 1).^17^ The early as well as more recent immunotherapies were all first approved in the adjuvant setting (given after tumor resection/main treatment). However, immunotherapies have been increasingly shown to demonstrate even greater potency in the neoadjuvant setting (given before tumor resection/main treatment).^18−21^ Unlike neoadjuvant cytotoxic chemotherapy, which has as its primary advantage the ability to debulk a tumor to enable surgery, neoadjuvant immunotherapy takes advantage of increased tumor antigen presence to enable a more potent immune response prior to surgery that persists after primary tumor resection. As one example, neoadjuvant immunotherapy with the immune checkpoint inhibitors ipilimumab and nivolumab resulted in greater expansion of tumor-related T cell clonotypes in peripheral blood than seen with adjuvant immunotherapy in the OpACIN clinical trial.^22^

Taken together, over a widening number of cancer types and stages, there is an increasing number of immunotherapy-eligible patients who may possess a primary tumor at the time of the first immunotherapeutic treatment and retain at least some degree of intact lymphatic vasculature and TDLNs. Tumor-lymphatic crosstalk is thus clinically relevant not only in terms of disease progression (e.g., metastasis) but also for therapeutic manipulation of the ongoing antitumor immune response. Deliberate modulation of this crosstalk has the potential to generate therapeutic responses both locally and systemically to control both treated lesions and untreated metastases. In this light, this review will elaborate on the nature and qualities of immune system crosstalk facilitated by the tumor-lymphatic tissue interface, detail therapeutically actionable strategies related to these pathways, and highlight opportunities for engineered drug delivery systems to favorably manipulate this crosstalk in the context of intratumoral immunotherapy to improve treatment efficacy and therapeutic outcomes. Previous cancer immunotherapy reviews have discussed the biology of immune cell crosstalk in the tumor, therapeutic pathways for intratumoral immunotherapy, the biology of immune cell crosstalk in the TDLN, and possibilities for TDLN-targeted immunotherapy, but these areas have generally been considered as largely distinct domains rather than viewed as part of a unified tumor/lymphatic therapeutic strategy.^1,6,23,24^ Similarly, while previous reviews on drug delivery have discussed possibilities for engineered drug delivery systems to mitigate toxicities from intratumoral immunotherapy, to improve therapeutic retention, and to engineer therapeutic delivery to the TDLN, they have not explicitly connected these important questions to an overall goal of simultaneous therapeutic immunomodulation of the tumor and lymphatic system.^25−29^ This review synthesizes the emerging clinical immunotherapeutic paradigm with the rationale for therapeutic modulation of the tumor and lymphatic system, key pathways with potential for simultaneous modulation in the tumor and tumor-associated lymphatics/TDLNs, and modalities and drug delivery technologies relevant to these shared key pathways as a strategy for improving the treatment efficacy and therapeutic outcomes of intratumoral immunotherapy.

## 2 Tumor-Lymphatic Immune Crosstalk

**Figure 2:**
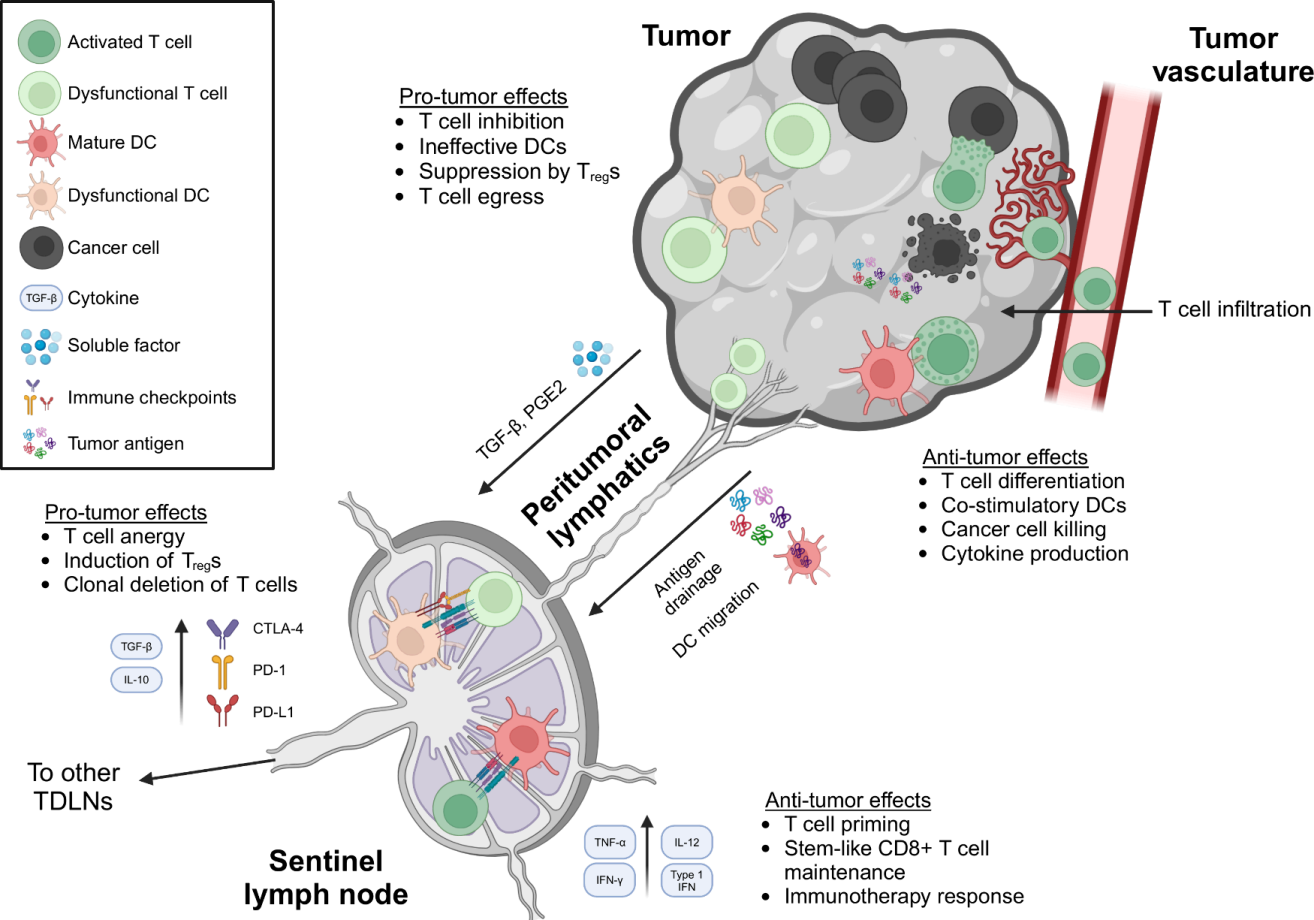
Tumor-lymphatic immune crosstalk involves an interplay between antitumor and pro-tumor immune effects in the lymphatics and TDLNs. TDLNs facilitate an intersection between tumor antigen, DCs, and T cells, promoting antitumor effects including tumor-reactive T cell priming and the formation and maintenance of an immunotherapy-responsive stem-like T cell niche (bottom right). These effects translate to antitumor effects within the tumor itself, culminating in effective cancer cell killing. At the same time, tumors can coopt lymphatics and TDLNs in order to orchestrate pro-tumor effects, increasing immune checkpoint expression and suppressive cytokine secretion as well as promoting DC dysfunction and T cell anergy, resulting ultimately in tumor escape (top left).

TDLNs, especially but not limited to the SLN(s), play a critical role in the establishment and maintenance of antitumor immunity (Figure 2). Lymph containing tumor antigen as well as other tumor-derived factors continuously drain from the tumor microenvironment and surrounding tissue interstitium via lymphatic capillaries to TDLNs. There, APCs such as dendritic cells (DCs) that reside in the LN sample lymph-draining antigen for processing into peptides and presentation on major histocompatibility complex (MHC) molecules. Alternatively, migratory DCs take up antigens in the tumor microenvironment before migrating to the TDLN via lymphatic vessels. DCs presenting tumor antigen can then interact with T cells to present antigen for T cell priming as well as providing important secondary signals such as costimulation through the CD28-B7 axis and proinflammatory cytokines to initiate effective T cell activation and proliferation.^30−32^ Critically, this behavior appears restricted to TDLNs, as recent work has demonstrated that, while T cells can differentiate in the tumor via interacting with APC niches there, tumor-reactive T cells undergo priming and clonal expansion in the TDLNs but not substantially in the tumor or in other lymphoid tissues such as noninvolved lymph nodes or the spleen.^33,34^ Because TDLNs are the primary sites for tumor-reactive T cell activation and expansion, they form the primary reservoir from which antigen-experienced tumor-reactive T cells enter the bloodstream and traffic to the tumor microenvironment, enabling tumor control. Notably, this characteristic of TDLNs is directly impacted by upstream lymphatic function: research in both mice and humans has linked higher relative lymphatic gene expression in the tumor to increased tumor infiltration by lymphocytes.^2,35^

TDLNs are also notable in that they maintain an immunotherapy-responsive T cell pool. Seminal work from multiple groups has demonstrated that stem-like CD8+ T cells, an antigen-experienced CD8+ T cell subset that retains the capacity for both self-renewal and further differentiation, are the primary CD8+ T cell subset that responds to therapeutic immune checkpoint blockade (ICB).^36−38^ Subsequent work has demonstrated that a niche of these stem-like CD8+ T cells is maintained in TDLNs throughout tumor progression, in part due to the role played by migratory DCs transiting from the tumor microenvironment to the lymph node via the peritumoral lymphatics.^39,40^

TDLNs have also been implicated in mediating the immune response to and the abscopal effect generated by tumor-directed radiotherapy, which can in some ways be thought of as a classical example of intratumoral therapy. For example, tumor-directed radiotherapy has been shown preclinically to generate an abscopal effect that correlates with enhanced CD8+ T cell proliferation in the TDLN, but only if the TDLN itself is spared irradiation.^41^ The generation of potent immune responses in TDLNs, then, is important for eliciting systemic immunity and treating distant tumors. Similar findings have corroborated this in humans, with an immunostimulatory effect observed in the TDLN in response to low-dose radiation which is abrogated when using high-dose radiation.^42^ This implies that an enhanced antitumor immune response to intratumoral immunotherapy is to some degree contingent upon intact peritumoral lymphatics and TDLNs.

Despite the critical roles played by the TDLNs and peritumoral lymphatics in generating and maintaining the antitumor immune response, the presence of a tumor exerts substantial suppressive effects on the lymphatics and on TDLNs (Figure 2). One well-documented mechanism for this is the way in which tumors can bias DCs toward a tolerogenic phenotype, resulting in dysfunctional or anergic T cell priming in the TDLN. For example, in both mice and humans, tumor-derived soluble factors such as transforming growth factor beta (TGF-β) and prostaglandin E2 (PGE2) reduce MHC and costimulatory ligand expression and enhance immune checkpoint ligand expression on DCs in the TDLN.^43,44^ This has functional consequences for T cell antitumor activity including increased CD8+ T cell anergy and induction of suppressive CD4+ regulatory T cells (T_reg_s).^45,46^ The immunosuppressive cytokine IL-10 is also known to be increased in TDLNs with downstream consequences for TDLN-resident APCs such as reduced costimulatory ligand expression and downregulation of proinflammatory cytokines like IL-12.^47^ Similarly, tumor-derived M-CSF, GM-CSF, and IL-6 promote myeloid-derived suppressor cell (MDSC) recruitment to and expansion in the TDLN.^48^

In addition to suppressive effects exerted by the tumor on APCs, tumor presence can act on T cells without intermediaries in order to inhibit T cell function and promote suppressive T cell phenotypes in the TDLN. In addition to its well-documented effects on APCs, tumor-derived TGF-β can also directly inhibit T cell differentiation and function.^49,50^ Furthermore, the TDLN sees preferential differentiation of tumor antigen-specific CD4+ T cells into T_reg_s over effector T cells, even at early stages of tumor growth.^51^ Perhaps most notoriously, tumor presence is associated with increased expression of immune checkpoints in the TDLN. Upregulation of the immune checkpoints programmed cell death protein (PD) 1 and programmed death ligand (PD-L) 1 on immune cells in the TDLN compared to other tissues and consequent increases in immune suppression of T cell function have been extensively demonstrated.^7,52^ Interaction between PD-1 on T cells with its ligands, PD-L1 (expressed on a wide variety of cells including APCs and cancer cells) and PD-L2 (expressed primarily on APCs) affects T cell signaling downstream of the T cell receptor (TCR) and costimulatory receptor CD28 to promote T cell inhibition as well as disrupting TCR-CD8 cooperativity during antigen recognition.^53,54^ Similarly, cytotoxic T lymphocyte associated protein 4 (CTLA-4), which limits T cell activation by outcompeting CD28 for costimulatory ligand binding, is upregulated in the TDLN. Whereas PD-1/PD-L interactions suppress T cell function in both the TDLN and the TME, CTLA-4 is understood to be primarily active in the TDLN due to its role in regulating T cell priming.^55^ Additional research has shown increased expression of other immune checkpoints in the TDLN as well, such as lymphocyte activation gene 3 (LAG-3), which prevents CD4+ T cells from interacting efficiently with MHC-II.^55,56^

Beyond merely exerting effects on immune cells in the TDLN, tumors can also modulate the lymphatic endothelial cells (LECs) that make up lymphatic vessels and LN sinuses through diverse mechanisms to decrease antitumor immune activity. Part of this modulation involves the induction of passive mechanisms of immune suppression or tolerance. For example, while LECs constitutively express PD-L1 as part of their role in maintaining immune homeostasis, PD-L1 is upregulated in tumor-associated lymphatic vessels in comparison with nonassociated lymphatic vessels with the result that T cells interacting with these vessels are suppressed.^57^ LECs can also be coopted to play an active role in tumor-induced immune suppression: tumor-derived vascular endothelial growth factor C (VEGF-C) induces LECs in the TDLN to promote the deletion of tumor antigen-specific CD8+ T cells.^58^ LECs can affect T cell behavior once T cells have migrated to a tumor site, as well. Multiple groups have demonstrated that, in mice, T cells can continuously egress the tumor via peritumoral lymphatics to the TDLN.^59,60^ Recent investigation into this phenomenon has shown that the LECs that comprise peritumoral lymphatics upregulate CXCL12, influencing how different qualities of T cell are either retained at the tumor site or induced to egress the tumor.^61^

The importance of the TDLN and peritumoral lymphatics in mediating both pro-tumor and antitumor immune activity motivates the use of therapeutic approaches that modulate these pathways. Because intratumoral immunotherapy can simultaneously enable greater drug accumulation in the tumor and increase drug access to the tumor lymphatic basin compared to systemic administration, it has a unique potential to simultaneously modulate tumor-lymphatic crosstalk in the tumor, peritumoral lymphatics, and TDLN.

## 3 Therapeutically Relevant Targets and Pathways for Intratumoral Immunotherapy

### 3.1 Oncolytic Viruses

To date, oncolytic viruses are the only intratumoral immunotherapy to receive FDA approval, making them particularly worthy of discussion here. Oncolytic viruses infect and kill cancer cells, inducing immunogenic cell death and releasing tumor antigen and damage-associated molecular patterns (DAMPs), as well as pathogen-associated molecular patterns (PAMPs) from the virus itself, to spur an antitumor immune response.^62^ Talimogene laherparepvec (T-VEC) is an oncolytic herpes virus that transduces infected cells with a gene encoding the cytokine GM-CSF in order to enhance DC recruitment to and maturation within the tumor and is cleared for intratumoral treatment of recurrent unresectable cutaneous, subcutaneous, and nodal melanomas in the adjuvant setting. More recently, T-VEC has also been investigated in the neoadjuvant setting, demonstrating somewhat improved relapse-free and overall survival vs resection alone (Table 1).^63^ The success of T-VEC has inspired extensive clinical efforts toward oncolytic viruses, and T-VEC itself may hold important design cues for subsequent effective oncolytic viruses. Notably, the therapeutic effects seen in response to T-VEC are likely due to a combination of immunogenic cell death and other immunomodulatory effects rather than direct viral cancer cell killing.^64^ Investigations into patients receiving T-VEC showed that frequencies of suppressive T_reg_s and MDSCs were decreased in treated lesions compared to nontreated lesions and that nontreated lesions had increased infiltration by CD8+ T cells post-treatment, suggestive of local and potentially also systemic antitumor immune responses.^65,66^ However, T-VEC has not yet been shown to significantly improve overall survival vs standard of care or have clinically significant effects on distant metastases to tissues such as the lung or the liver.^67^ Despite this, as the only FDA-approved intratumoral immunotherapy, it sets a reasonable threshold for efficacy that other intratumoral immunotherapies must meet if not exceed.

**Table 1:** Selected Recent Neoadjuvant Intratumoral Immunotherapy Clinical Trials

Trial Number	Trial Stage	Cancer Types	Disease Stage	Intervention	Effect
NCT02211131	Phase II	Melanoma	Stage III/IV	Oncolytic virus (T-VEC)	Improved relapse-free and overall survival vs resection alone
NCT02274155	Phase I	HNSCC	Stage III/IV	aOX40 mAb (MEDI6469)	T cell proliferation in peripheral blood, increased activated TILs
NCT02706353	Phase I/II	Melanoma	Stage IV	aCD40 mAb (AMX005M) with i.v. aPD-1 mAb (pembrolizumab)	Increased antigen presentation locally, increased T cell activation and clonal diversity locally and in distant lesions (vs aPD-1 alone)
NCT02379741	Phase I	Solid tumors	Stage IV	aCD40 mAb (mitazalimab)	Increased CD86+ B cells in peripheral blood
NCT06205849	Phase I	Pancreatic cancer	Stage III	aCD40 mAb (mitazalimab)	Ongoing
NCT02857569	Phase I	Melanoma	Stage IV	aCTLA-4 mAb (ipilimumab) with i.v. aPD-1 mAb (nivolumab)	Lower toxicity i.v. vs i.t., depletion of intratumoral T_regs_ for i.t. only
NCT04695977	Phase II/III	Melanoma	Stage III/IV	TLR9 agonist (vidutolimod) with i.v. aPD-1 mAb (nivolumab)	Ongoing
NCT03262103	Phase I	Prostate cancer	Stage I/II	TLR3 agonist (Hiltonol)	Increased TIL density in resected sample compared to pretreatment biopsy, TLS formation, increased T cell/NK cell response in peripheral blood
NCT06343077	Phase II	Prostate cancer	Stage I/II	TLR3 agonist (Hiltonol)	Ongoing
NCT06022029	Phase I	Solid tumors and lymphomas	Stage III/IV	STING agonist (ONM-501) with i.v. aPD-1 mAb (cemiplimab)	Ongoing
NCT04799054	Phase I/II	Solid tumors	Stage III/IV	TLR7/8 agonist (resiquimod) with i.v. aPD-1 mAb (pembrolizumab)	Ongoing
NCT02076633	Phase II	Melanoma	Stage III/IV	IL-2 and TNF fused to antifibronectin mAb (Daromun)	Abscopal responses in noninjected lesions
NCT02938299	Phase III	Melanoma	Stage III	IL-2 and TNF fused to antifibronectin mAb (Daromun)	Improved recurrence-free survival vs resection alone
NCT03739931	Phase I	Solid tumors and lymphoma	Stage III/IV	mRNA encoding OX40L, IL-23, IL-36y (mRNA-2752) with i.v. aPD-L1 mAb (durvalumab)	Ongoing
NCT05539157	Phase I	Solid tumors	Solid tumors	mRNA encoding IL-12 (JCXH-211)	Increased T and NK cell infiltrate in treated lesions, observed abscopal response in 1 patient
NCT06171750	Phase 1	Solid tumors	Stage III/Stage IV	Aluminum hydroxide-anchored IL-12	Ongoing

Clinical investigations of oncolytic viruses have often neglected to examine in depth the effects occurring in the SLN (or other TDLNs if the patient has undergone SLNB) and consequently oncolytic virus candidates may not be optimized to elicit immunomodulatory effects in both the tumor and the LN. One possibility to enhance the effects of oncolytic viruses is to seek to engineer the virus to drive greater antigen, DAMP, and PAMP release in order to promote a stronger antitumor immune response both in the tumor and in the TDLN. This can in turn drive increased DC and T cell recruitment to the treated tumor while also enhancing antigen and DAMP/PAMP drainage to the TDLN, facilitating greater T cell priming and increasing the pool of tumor-reactive T cells with the potential to generate a systemic antitumor T cell response. Combining an optimized oncolytic virus with other tumor- and TDLN-targeted approaches such as locoregional ICB might generate substantially improved therapeutic effects.

### 3.2 Immunomodulatory Antibodies

#### 3.2.1 Targeting Immune Checkpoints in the Tumor and in the TDLN

Since their development, therapeutic antibodies have been an important modality for cancer therapy, and their relevance has increased only since the dawn of ICB therapy as a mainstay of medical oncology. While their obvious advantages include antigen specificity and long circulation times due to their ability to engage with neonatal Fc receptors, an interesting additional feature of therapeutic IgG monoclonal antibodies (mAbs) relevant to this discussion is that their size (∼150 kDa) is highly amenable to lymphatic uptake and drainage to the TDLN via peritumoral lymphatics.^68,69^ Consequently, targeting T cells in the tumor with intratumoral antibodies can enable simultaneous targeting of T cells in the TDLN. Interestingly, ICB mAbs currently marketed as systemic therapies are being reformulated for locoregional delivery, with a subcutaneous version of the aPD-L1 antibody atezolizumab receiving EMA approval in 2023 after showing noninferior drug exposure and comparable efficacy and safety to intravenous atezolizumab and similar results for a subcutaneous version of the aPD-1 antibody nivolumab.^70,71^ While these formulations are administered subcutaneously rather than intratumorally and are not designed to target TDLNs specifically, they represent a conceptual step toward the clinical realization of intratumoral antibody administration relevant to this discussion.

The rationale for ensuring delivery of ICB to the TDLN has been extensively validated in recent years. Preclinical research has demonstrated that the TDLN is primarily responsible for the initial immune response to ICB (whether aPD-1, aPD-L1, or aCTLA-4), and that administering ICB mAbs to T cells in the TDLN alone has similar immunomodulatory and therapeutic effects to administering ICB to T cells in the TDLN and in the tumor.^52,68^ Early in-human data has confirmed the importance of the SLN in ICB response, with patients who received a single low dose of the aCTLA-4 antibody tremelimumab at the tumor resection site before SLNB exhibiting increased effector T cell activation and reduced T_reg_ presence in both the SLN and peripheral blood, enhanced antigen-specific peripheral blood T cell response upon *ex vivo* restimulation, and even enhanced migratory DC presence in the SLN.^72^ Consequently, intratumoral administration of ICB mAbs, which can increase T cell exposure to mAb in the TDLN as well as in the tumor, has significant therapeutic potential. In addition, in light of the upregulation of PD-L1 on LECs in peritumoral lymphatics and in the TDLN, intratumoral administration of aPD1 or aPD-L1 has the potential to mitigate the tumor-induced modulation of T cell activity by LECs. Intratumoral administration is particularly interesting for targeting immune checkpoints, like CTLA-4, whose inhibition has been well documented to show dose-dependent antitumor effects but also dose-dependent toxicities.^73,74^ The ability of intratumoral mAb injection to potentially increase mAb localization to the TME and lymphatic basin and decrease systemic tissue exposure can help navigate this complexity.^75^

#### 3.2.2 TCR Agonism and Costimulatory Receptor Agonism in the Tumor and in the TDLN

Agonistic antibodies against T cell costimulatory receptors have been a difficult area of exploration ever since the serious adverse events seen in the clinical trial of the anti-CD28 superagonist TGN1412.^76^ Providing effective costimulation to T cells independently of APCs has potential to generate significant T cell antitumor responses, but because costimulatory mAbs are typically given systemically, they tend to come with major side effects and may be dose-limited at a subeffective dose.^77,78^ Increasingly, there is increased interest in administering these modalities intratumorally rather than systemically. One T cell costimulatory receptor that has attracted significant attention both preclinically and clinically is the tumor necrosis factor (TNF) superfamily member OX40, which has a variety of roles including enabling T cell survival and regulating cytokine production.^79^ After preclinical reports described the potent antitumor effects of intratumoral OX40 agonism in mice, there are now ongoing early investigations exploring the intratumoral administration of OX40 agonists in humans.^80,81^ Other costimulatory receptors such as 4-1BB (CD137) have shown promising effects in response to intratumoral agonism in preclinical work but have not yet been investigated for intratumoral administration in the clinic.^82,83^

Another interesting therapeutic pathway that has been successfully utilized in treating hematological malignancies and that is now increasingly being investigated for solid tumors is bispecific T cell engagers (BiTEs), with a recent FDA approval in uveal melanoma for tebentafusp-tebn. BiTEs are antibodies or fusion proteins containing both a CD3-binding domain and a tumor antigen-binding domain, such as CD19 for B cell malignancies or EpCAM for solid tumors, that can induce T cell activation upon the engagement of both domains.^84^ Despite BiTEs’ documented clinical success and increasing investment into developing them for solid tumors, clinical investigations into intratumorally administered rather than systemically administered BiTEs remain lacking. Preclinical investigation into intratumoral administration of oncolytic viruses encoding BiTEs, however, has demonstrated promising results.^85,86^ Particularly relevant for further investigation might be the intratumoral administration of larger BiTEs (formatted as bispecific antibodies or as fusion proteins with Fc domains) capable of lymphatic drainage to target metastases within LNs. In a similar vein, a recent preclinical study reported that the intratumoral delivery of plasmid encoding agonistic anti-CD3 mAbs in combination with IL-12 drives potent antitumor immunity, lending further credence to the idea of targeted TCR agonism via intratumoral administration.^87^

#### 3.2.3 CD40 Agonism to Activate Dendritic Cells

Agonism of CD40 on DCs, which leads to upregulation of MHC and costimulatory ligands, has been shown to be important for effective antitumor T cell responses.^88^ The presence of both resident and migratory DCs in the tumors presents a natural target for CD40 agonism. Additionally, intratumoral administration of aCD40 mAbs has been shown preclinically to result in increased aCD40 accumulation in TDLNs and consequent stimulation of LN-resident or recently migrated DCs to expand T cells there.^89,90^ In light of this, there have been some early investigations of intratumoral CD40 agonism in humans, with a recent clinical trial investigating the mAb APX005M resulting in increases in T cell activation gene signatures and clonal diversity in both treated and nontreated lesions, pointing toward the possibility for this approach to generate both local and systemic antitumor T cell responses (NCT02706353).^91,92^

#### 3.2.4 Using Therapeutic Antibodies to Engineer Targeted T_reg_ Depletion

Some therapeutic antibodies have Fc-dependent immunomodulatory effects in addition to immunomodulatory effects resulting from targeted agonism or antagonism. In mice, treatment with certain clones of aCTLA-4 mAbs can deplete intratumoral T_reg_s, enhancing the antitumor immune response. However, examination of clinical data has revealed that neither of the two FDA-approved aCTLA-4 mAbs (ipilimumab and tremelimumab) result in significant intratumoral T_reg_ depletion in humans in their current clinical context of systemic administration, but only when administered intratumorally, further motivating the use of intratumoral aCTLA-4.^75,93^ At the same time, because preclinical studies have indicated that aCTLA-4 mAbs are most effective when both their Fc-independent and Fc-dependent functions are engaged, engineering aCTLA-4 mAbs capable of better depleting intratumoral T_reg_s in humans has attracted substantial interest. For example, gotistobart, an aCTLA-4 mAb that is designed to enhance T_reg_ depletion in the tumor, is currently in phase 3 trials but is designed for systemic administration (NCT05671510). Conceivably, even next-generation aCTLA-4 antibodies like this one could benefit from intratumoral administration in order to increase the mAb concentration and T_reg_ depletion in the tumor and TDLN while seeking to minimize T_reg_ depletion in off-target tissues. Another example of a therapeutic antibody capable of depleting T_reg_s is a OX40. Preclinical data indicate that intratumoral aOX40 administration, in addition to its stimulatory effects on T cells via OX40 agonism, causes activating Fc receptor-dependent T_reg_ depletion in the tumor, further underscoring the opportunities available to engineer therapeutic antibodies to simultaneously stimulate effector T cells while also depleting T_reg_s following intratumoral administration.^80^

### 3.3 Immunomodulatory Small Molecules and Oligonucleotides

Pattern recognition receptor (PRR) agonists including toll-like receptor (TLR) agonists and stimulator of interferon genes (STING) agonists have potent immunostimulatory effects on APCs, increasing MHC and costimulatory ligand expression and inducing proinflammatory cytokine production. Intratumoral administration of PRR agonists may be critical for their success: not only do these agents see limited tumor and LN access from systemic administration, but because many of these modalities are nucleic acids, they are subject to rapid degradation *in vivo*. Indeed, early clinical trials of systemically administered TLR agonists were unsuccessful in spurring significant therapeutic responses at acceptable levels of toxicity. Intratumorally administered PRR agonists, on the other hand, have generated promising preclinical data indicating their ability to modulate immune responses in both the tumor and TDLN. Recent examples of this have included TLR3 agonists, dual TLR7/8 and RIG-I agonists, and STING agonists.^94−98^ This in turn has prompted a degree of interest in intratumoral administration of PRR agonists in humans, including agonists against TLR3, TLR7/8, or TLR9, and STING agonists, with some early reports indicating promising results such as increased T cell infiltration and TME remodeling in treated tumors and signs of systemic T cell response (NCT04695977, NCT06343077, NCT06022029, NCT04799054).^99^ However, it remains to be seen whether intratumoral administration of these drugs without coupling them with an engineered delivery system will consistently induce therapeutic responses. Despite preclinical evidence suggesting immunostimulatory effects in the TDLN downstream of effects in the tumor, PRR agonists themselves may be limited in their ability to consistently access peritumoral lymphatics and the TDLNs, for reasons discussed in later sections, at least without undesirable systemic effects being simultaneously elicited. Given the significant immunosuppressive effects within the tumor microenvironment, delivery of PRR agonists to APCs within the tumor alone may be insufficient to consistently induce potent antitumor immunity in humans and novel drug delivery approaches may be necessary in order to achieve therapeutically relevant delivery to both the tumor and the TDLN.

### 3.4 Immunostimulatory Cytokines

Cytokines have been of great interest for cancer immunotherapy due to their potent immunomodulatory effects. Indeed, some of the earliest FDA-approved cancer immunotherapies were cytokines, with the approvals of interferon (IFN) alpha for high-risk melanoma and high-dose interleukin (IL) 2 for metastatic renal cell carcinoma and metastatic melanoma. However, while systemically administered cytokines can spur durable responses, only a small fraction of patients respond and at the cost of significant toxicities such as capillary leak syndrome.^25^ Intratumoral cytokine administration can potentially increase drug accumulation in the TME and tumor-associated lymphatics to improve therapeutic effects while also reducing systemic exposure, and consequently has been an increasingly attractive option.^100^ This approach has recently seen some success in humans with the combination neoadjuvant therapy daromun, which consists of the cytokines IL-2 and TNF fused to tumor-targeting antibody L19 to enhance tumor retention. Intratumoral daromun treatment induced abscopal effects in nontreated tumors in a phase 2 trial and improved recurrence-free survival in a phase 3 trial (NCT02938299).^69,101^ A variation on this approach that has also garnered significant attention is intratumorally administering mRNA that encodes cytokines rather than administering the cytokines themselves. Recent preclinical work, for example, has explored the possibility of using intratumorally administered mRNA encoding a fusion protein combining IL-12 with a CD137 agonist and a TGF-β antagonist and observed both therapeutic effects in the treated tumor as well as abscopal effects.^102^ Similar approaches are currently undergoing investigation in humans, including a trial with mRNA encoding IL-23 and IL-36γ along with OX40 ligand and a trial with mRNA encoding IL-12 (NCT03739931, NCT05539157). Promisingly, previous in-human data have indicated that intratumoral cytokine treatment results in changes in the lymphocyte compartment in the TDLNs as well as in the tumor, highlighting the opportunities for intratumoral cytokines to modulate immune crosstalk in the TDLN as well as the injected tumor.^103^

### 3.5 Opportunities for Intratumoral Combination Therapy

Notwithstanding the opportunities presented by various classes of intratumoral immunotherapy in isolation, the greatest therapeutic potential may lie in exploring combinations of these modalities in order to provoke synergistic effects while simultaneously mitigating the toxicities that might be seen with the same combinations administered systemically. Possible approaches could include, for example, simultaneously enhancing immunogenic cell death (e.g., via an oncolytic virus) and providing T cell costimulation (aOX40) or stimulating APCs (PRR agonist) and blocking T cell inhibition while depleting T_reg_s (aCTLA-4). While there has yet to be extensive clinical investigation along these lines, recent preclinical data have helped underscore this possibility. One group demonstrated preclinically that combination intratumoral administration of a TLR9 agonist and OX40 agonist substantially enhanced the T cell response and antitumor activity in primary and metastatic tumors as well as in TDLNs.^104^ Others have shown marked tumor reduction in response to the combination of aCTLA-4, aCD137, and aOX40 as a result of substantial antibody accumulation in both the tumor and the TDLN, while still others have demonstrated significant immunomodulatory effects in both the tumor and TDLN arising following coadministration intratumorally of a TLR3 agonist and a STING agonist.^105,106^ Similarly, recent preclinical work has shown that intratumoral TLR9 agonist and IL-12 with or without aOX40 results in profound effects in the tumor and TDLN as well as abscopal effects.^107^ Because of the difficulties inherent in achieving effective drug accumulation in the tumor and TDLN even in the context of intratumoral administration, however, these therapeutic strategies can benefit from using engineered drug delivery systems (DDSs) that are optimized for intratumoral immunotherapy.

## 4 Engineering Drug Delivery Systems for Intratumoral Immunotherapy

**Figure 3:**
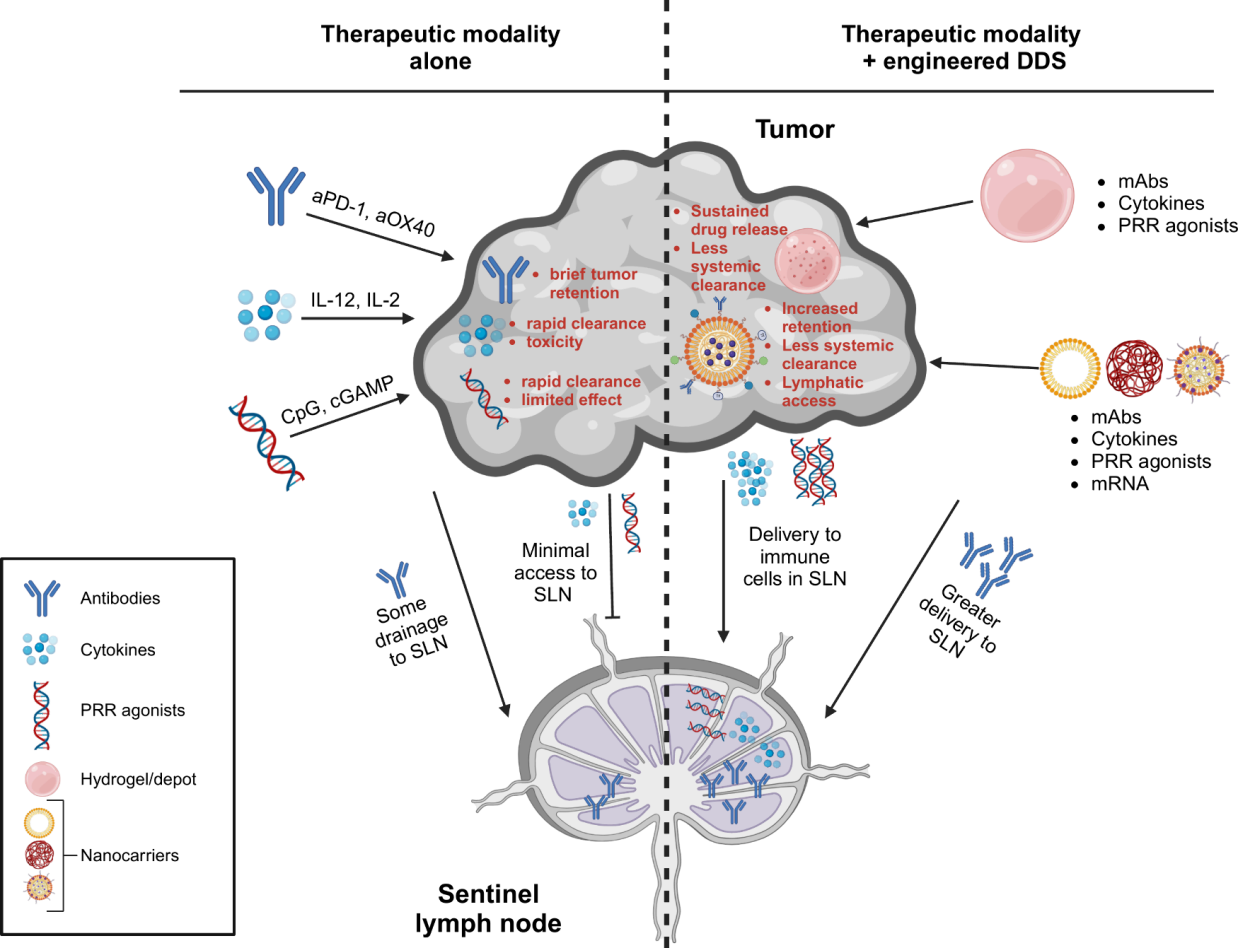
Engineered drug delivery systems improve the ability of intratumoral immunotherapy to modulate tumor-lymphatic immune crosstalk by improving drug access to and effects in both the tumor and the TDLN. Intratumorally administered therapies by themselves can transiently modulate immunity in the tumor but struggle to effectively access the lymphatics/TDLN, with antibodies seeing some accumulation but other modalities such as PRR agonists and cytokines seeing minimal LN access (left). Engineered drug delivery systems such as depot-forming/hydrogel systems and nanocarrier-based systems (right) can simultaneously enhance intratumoral immunotherapy access to tumor lymphatics/TDLNs to amplify antitumor immunity in those tissues as well as improve the effects in the tumor (e.g., by reducing rates of drug clearance).

An important factor to consider in the design of intratumoral immunotherapies to modulate tumor-lymphatic crosstalk is the extent to which a drug will reach the target tissues and target cell populations necessary for maximum efficacy. This consideration can be seen with the radioactive tracers typically used for lymphatic mapping, which are typically between 10 and 200 nm in diameter to prevent rapid clearance from the site of injection and enable lymphatic drainage and LN accumulation.^108^ On one hand, for modalities like oncolytic viruses it may suffice to achieve drug delivery primarily in the injected tumor and not in the TDLN, provided that the virus is sufficiently engineered to drive the release of antigen and DAMPs/PAMPs that can then drain via lymphatics. On the other hand, modalities like PRR agonists and some cytokines have significant direct effects both in the tumor and in the TDLN but are also relatively small in size (<5 nm hydrodynamic diameter). Cargoes in this size range do not see appreciable lymphatic exposure and are rapidly cleared from the tumor following intratumoral administration—meaning the administration of the drug by itself may be insufficient to achieve optimal immunomodulation in both the tumor and the lymphatic system (Figure 3).^109,110^ This dilemma highlights the opportunity to use engineered DDSs that can be tuned to achieve drug exposure in the TDLN as well as in the tumor to maximize therapeutic effects while also reducing systemic exposure and related toxicities.

### 4.1 Locoregional Sustained-Release and Depot-Forming Systems

Sustained-release or depot-forming systems represent one potential answer to the question of how to reduce systemic drug clearance after intratumoral administration and can be further manipulated to enhance the drug uptake by lymphatics. Many of these approaches involve developing hydrogels from biocompatible materials such as alginate or Pluronic that have desirable properties such as undergoing sol–gel transitions at physiological temperatures after drug loading and can be safely administered intratumorally. When applied to therapeutic antibodies such as ICB mAbs, these systems not only can enable increased antibody retention at the tumor site but also can sustain antibody access to the TDLN, whether passively by shifting the balance of antibody blood clearance vs lymphatic clearance in favor of the lymphatics or actively by shedding lymph-draining antibody-containing micelles.^111,112^ These systems can also be engineered to enhance the simultaneous tumor and lymphatic delivery of smaller drugs such as PRR agonists. For example, TLR agonist-containing polyamide-based nanocarriers capable of draining through lymphatic capillaries to be taken up by DCs in the TDLN were released from a polyethylene glycol (PEG)-based hydrogel that was modified to respond to reactive oxygen species.^113^ Additionally, hydrogels comprised of PEG-based microspheres to which a TLR agonist can be connected by a degradable linker have been developed, resulting in APC activation and T cell stimulation in both TDLNs and tumors.^114^ Notably, hydrogels can also be manipulated to deliver multiple classes of drug simultaneously, such as PRR agonist and antibody or PRR agonist and cytokine.^115,116^ Another noteworthy approach involves tethering the therapeutic modality of interest to a depot-forming system to improve drug retention at the tumor site. For example, IL-12 tethered to the depot-forming vaccine adjuvant aluminum hydroxide enhanced immune activity in the tumor and resulted in enhanced T cell activity in TDLNs and is currently being evaluated in humans (NCT06171750).^117^

### 4.2 Nanocarriers

Nanocarriers that are optimally sized to enter peritumoral lymphatics and drain to TDLNs are another potential solution for efficiently modulating tumor-lymphatic immune crosstalk. A variety of approaches toward this goal have been explored, with some additionally achieving a degree of sustained release by balancing injection site retention and lymphatic drainage or successfully limiting systemic exposure by constraining nanocarrier/drug accumulation to the tumor and the TDLN. Various groups have used nanoparticles to intratumorally administer cargoes as diverse as antibodies, STING agonists, and TLR agonists. In doing so, they have demonstrated biodistribution that is primarily limited to the tumor, peritumoral lymphatics, and TDLN as well as immunomodulatory effects including DC maturation, T cell activation, and cytokine secretion in those tissues.^118−122^ Many of these efforts feature interesting design features to enhance targeted drug delivery. One such effort involved the synthesis of photosensitive and matrix metalloprotease-2-degradable PEG-based nanoparticles loaded with aPD-L1 that improved drug accumulation following irradiation not only in the primary TDLN but in TDLNs further downstream as well.^118^ Another involved the development of PEG/polyphosphoester-based nanoparticles loaded with TLR agonist that, when injected intratumorally after magnetic hyperthermia treatment, were capable of noncovalently adsorbing tumor antigen on the surface of the particle before draining to the TDLN thus in principle enabling simultaneous TLR agonist and antigen delivery to TDLN DCs.^120^ In another interesting approach, PEG- and poly lactic-*co*-glycolic acid (PLGA)-derived nanoparticles that were functionalized with tannic acid were formed, enabling the lymph-draining nanoparticles to enter LN conduits and penetrate deep into the LN to target T cells and DCs in the paracortex rather than arresting in the subcapsular sinus.^121^ A subset of nanocarrier work has investigated deploying biopolymers such as glatiramer acetate or glycol chitosan with intrinsic adjuvant properties for intratumoral immunotherapy. Formulations of these biopolymers can form particle solutions upon injection capable of both stimulating APCs in the TME and draining via lymphatics to stimulate APCs in TDLNs.^123,124^ Given the enormous wave of interest in liposomes and lipid nanoparticles (LNPs) leading up to but especially in the wake of the SARS-CoV-2 pandemic and the successful LNP-mRNA vaccines, there have also been recent forays into using LNPs for intratumoral administration of therapeutic cargoes including antibodies, cytokines, and STING agonists as well as mRNA encoding a variety of cargoes such as cytokines, costimulatory ligands, and novel fusion proteins.^102,125−128^ Intratumorally administered mRNA LNPs are particularly interesting for their potential to turn immune cells in both the tumor and the TDLN into factories producing specific immunostimulatory antibodies or cytokines, in contrast to intratumorally administered naked mRNA which results in protein translation in the tumor but not the TDLN.^127,128^ Particularly interesting will be in-human data indicating the extent to which intratumorally administered LNPs are able to mitigate the liver tropism for which LNPs are generally known.^129^ Another interesting development has been the functionalization of nanocarriers, such as polymeric nanoparticles or LNPs with agonistic aCD3 antibodies or binding domains from aCD3 antibodies. Administration of such particles can not only stimulate T cells but can also deliver cargoes like mRNA or small molecule drugs to those same T cells and could enable highly specific T cell targeting in the tumor and TDLNs.^130,131^

## 5 Potential Future Directions for Intratumoral Immunotherapy

Many exciting opportunities are emerging for intratumoral immunotherapy to modulate tumor-lymphatic immune crosstalk. Approaches using DDSs to deliver combinations of immunomodulatory therapies (e.g., cytokine and PRR agonist, cytokine and mAb, PRR agonist and mAb) to both the tumor and the lymphatic system have the potential to overcome some of the limitations of monotherapies, though a clearer understanding of potential toxicities from intratumoral combination therapy may be necessary before clinical success is achieved. Given the increasing interest in developing BiTEs for solid tumors, there may also be value in pursuing the intratumoral administration of BiTEs either in protein form or encoded by oncolytic virus to target both the tumor and TDLN, along with other forms of therapeutic intratumoral TCR agonism.

Beyond the approaches detailed here, there are also newfound insights and developing therapeutic approaches that may be applied to an intratumoral immunotherapeutic paradigm. For example, therapies that remodel the TME and lymphatic system in order to enable increased T cell recruitment, infiltration, and retention in the TME require further investigation. Recent work has identified the formation of immunotherapy-inducible tertiary lymphoid structures (TLS) within the TME that enable increased T cell infiltration into and survival in the tumor.^132−134^ Various chemokines and cytokines, including CCL21, CXCL13, LIGHT, and lymphotoxin (LT) β, have all been implicated in TLS formation.^132,135^ There may be an opportunity to use DDSs to engineer enhanced TLS formation in the TME to synergize with other immunotherapies. For example, sustained-release or depot-forming systems could be loaded with recombinant LIGHT or agonistic LTβR mAbs to increase the level of T cell infiltration into the TME, potentially improving the effects of ICB. Immunomodulatory small molecules also represent a plausible use case for DDSs. Particularly interesting are inhibitors of the enzyme protein tyrosine phosphatase (PTP) that can enhance TCR and cytokine signaling in T cells and demonstrate significant antitumor potential. Inhibition of PTPN1 and PTPN2 has been shown to enhance CD8+ T cell activation, proliferation, and cytokine secretion in response to antigen stimulus *ex vivo* and to increase TIL density *in vivo*.^136−139^ Because systemic administration of these potent inhibitors in humans raises questions around safety and tolerability, modalities like these could also benefit from targeted delivery to tumors and TDLNs.

## 6 Conclusion

The intentional therapeutic modulation of not only the tumor but also the tumor-associated lymphatic system offers opportunities for augmenting antitumor immune activity to achieve curative therapeutic efficacy in advanced disease. To aid in this goal, the development of engineered DDSs to better enable the manipulation of tumor-lymphatic crosstalk is critical. In an emerging practice era characterized by less invasive LN biopsy and removal, as well as more immunotherapies being initiated in the neoadjuvant stage, previously overlooked opportunities in the design of intratumoral immunotherapies offer unique advantages to define new paradigms of cancer immunotherapy. While recent late-phase clinical trials of intratumoral immunotherapies in advanced or metastatic cancers have succeeded in generating promising local responses, most have yet to consistently achieve durable systemic benefit—likely due in part to failures to generate sufficiently potent systemic antitumor immune responses.^140^ Due to intratumorally administered immunotherapies offering both therapeutic effects within the injected tumor as well as access to LNs where systemically active antitumor immune responses can be modulated, opportunities exist to incorporate recent insights into tumor-lymphatic crosstalk and advances in DDSs in order to design intratumoral immunotherapies that, in addition to providing local control, also enhance and amplify systemic immunity.

## Abbreviations

LN: lymph node
TDLN: tumor-draining lymph node
CLND: complete LN dissection
SLNB: sentinel LN biopsy
TME: tumor microenvironment
APC: antigen presenting cell
DC: dendritic cell
MDSC: myeloid derived suppressor cell
ICB: immune checkpoint blockade
MHC: major histocompatibility complex
PD-1: programmed cell death protein 1
PD-L: PD-ligand
CTLA-4: cytotoxic T lymphocyte associated protein 4
LAG-3: lymphocyte activation gene 3
TCR: T cell receptor
IL: interleukin
TGFβ: transforming growth factor β
LEC: lymphatic endothelial cell
mAb: monoclonal antibody
BiTE: bispecific T cell engager
PRR: pattern recognition receptor
TLR: toll like receptor
STING: stimulator of interferon genes
PEG: polyethylene glycol
LNP: lipid nanoparticle
